# Ethical conflicts associated with COVID‐19 pandemic, triage and frailty—unexpected positive disease progression in a 90‐year‐old patient: A case report

**DOI:** 10.1002/ccr3.7710

**Published:** 2023-07-18

**Authors:** Andreas Wehrfritz, Joachim Schmidt, Frank Bremer, Anne‐Katharina Lang, Jacob Welzer, Ixchel Castellanos

**Affiliations:** ^1^ Department of Anaesthesiology University hospital of Erlangen, Faculty of Medicine, Friedrich‐Alexander‐University Erlangen Erlangen Germany; ^2^ Department of Anaesthesiology Klinikum Fuerth Fuerth Germany

**Keywords:** aged patient, COVID‐19, ethical dilemma, triage and frailty, unexpectedly positive trend

## Abstract

During the COVID 19 pandemic, advanced age, scoring systems, and a shortage of ICU beds were used as cut‐offs for ICU admission. This case report describes the epicrisis of an elderly patient who was almost mistakenly not treated in an ICU.

## Graphical abstract

Chest radiograph of a 90‐year‐old patient with bipulmonary infiltrates typical of COVID‐19, already covering 70% of the lung parenchyma. The unexpectedly positive disease course in this 90‐year‐old patient raises ethical conflicts related to the COVID‐19 pandemic, standard triage practice at the time, and the frailty score used for this purpose.

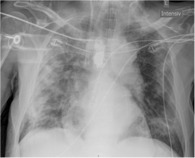

## 1 INTRODUCTION

In December 2019, the novel severe acute syndrome coronavirus (SARS‐CoV‐2) began its pandemic worldwide spread in the city of Wuhan in the public republic of China.^1^ This led WHO to declare a Public Health Emergency of International Concern on January 30, 2020, and to characterize the outbreak some weeks later as a pandemic on March 11, 2020.^2^ Beside droplet infection, aerosol transmission plays a major role for infections. All of the world's health care systems are struggling with a lack of resources, which became particularly acute in most countries during the COVID 19 pandemic.^3^ This was widely reported in the media, and depending on the economic status of a country and its healthcare system, one country or another was more or less spared from this resource shortage. Many countries therefore began to establish triage systems to ensure adequate medical care for as many patients as possible during this flood of patients during the pandemic.^4^, ^5^, ^6^, ^7^ It has been reported, that older adults living in community‐dwelling or nursing homes are not only the most vulnerable group with regard to infection but have also the highest mortality rates for COVID‐19 because of several comorbidities and age‐related immunosenescence.^8^, ^9^ Unfortunately, especially in the early days of the pandemic, a higher age of patients was used as a cut‐off for admission to an intensive care unit when the ICU resources are low. However, this cannot be a stand‐alone marker. To specify the health status of elderly patients, the result of the frailty score is used, but even this cannot be a complete decision guide and often represents only a definite short period of time.^10^ In particular, in patients who have suffered from physical and/or mental disabilities as childhood, this score is not validated in older age. Some studies have already discussed a possible ethical dilemma associated with the frailty score.^11^ The purpose of this case report is to point out that each patient should be considered individually and that although frailty may be the usual multifactorial endpoint, it is not necessarily so in every patient.

## 2 CASE PRESENTATION

Ten days after a positive reverse transcriptase polymerase chain reaction (RT‐PCR) test for SARS‐CoV2, a 90‐year‐old patient was admitted to the emergency department of our tertiary university hospital by his primary care physician with a rapid deterioration of his general condition and existing hypoxemia (SpO_2_ 77%) as well as acute symptoms, associated with the COVID‐19 disease. At this time, the patient had received his basic immunization with one COVID‐19 vaccine only 8 days before the onset of illness. Sequence analysis revealed the presence of variant B1.1.7. In the emergency department, he immediately received oxygen therapy with a high‐flow nasal cannula (HFNC) and immunomodulatory therapy with dexamethasone 6 mg according to the German S3 guidelines at that time. As a result of acute to chronic renal failure, an initial creatinine of 320.89 μmoL/L was detected. The patient had been suffering from persistent vomiting and diarrhea for several days and shows an additional existing hypernatremia (153 mmoL/L) and a lactate value of 1.8 mmoL/L.

The patient's medical history at that time included type II diabetes mellitus, atrial fibrillation, chronic renal insufficiency, previous cataract surgery of both eyes, and conservatively treated prostate carcinoma. The initial screening of the different scoring systems revealed the following results: Sequential Organ Failure Assessment (SOFA) 4, Simplified Acute Physiology II Score (SPAS‐II) 50, Therapeutic Intervention Scoring System (TISS‐28) 22, Charlson comorbidity index (CCI) 12, and Clinical Frailty Scale (CFS) 7. Due to an intrapartum rhesus incompatibility reaction, both the patient and his twin brother suffered from infantile hypoxic brain injury. During the Nazi regime in Germany, the parents succeeded only with difficulty in hiding both children from the then threatened “euthanasia” by the Nazi regime. In the CSF it is asked what abilities the person had 2 weeks ago, the current condition is not to be assessed, the person must be over 65 years old. There must always be a full assessment. It should be explicitly noted again that the CFS cannot be applied to individuals with early brain injury, therefore this score was not applied to this patient and is provided here only for completeness.

Since the 1960s, the patient and his twin brother have been living in a residential community for disabled people and taking an active part in life. Two weeks earlier, an outbreak of SARS‐CoV2 occurred in this nursing home, with 37 out of 62 residents testing positive to the virus. This outbreak resulted in seven hospitalizations with COVID‐19 pneumonia. Two residents required treatment in the intensive care unit, and one resident died. The patient's twin brother complained of only mild symptoms of diarrhea and nausea, which improved rapidly after symptomatic therapy.

Despite the patient's advanced age and his comorbidities was the patient's wish, which was brought by his niece who was caring for him, regarding on full intensive care treatment, including possible intubation, ventilation, hemofiltration, and veno‐venous extracorporeal membrane oxygenation (ECMO). Of course, we fully supported his will give his previously good quality of life and the repressions he had experienced during the Nazi regime. There was to be no euthanasia or triage based on biological age or disabilities that the patient had managed well since childhood.

**FIGURE 1:**
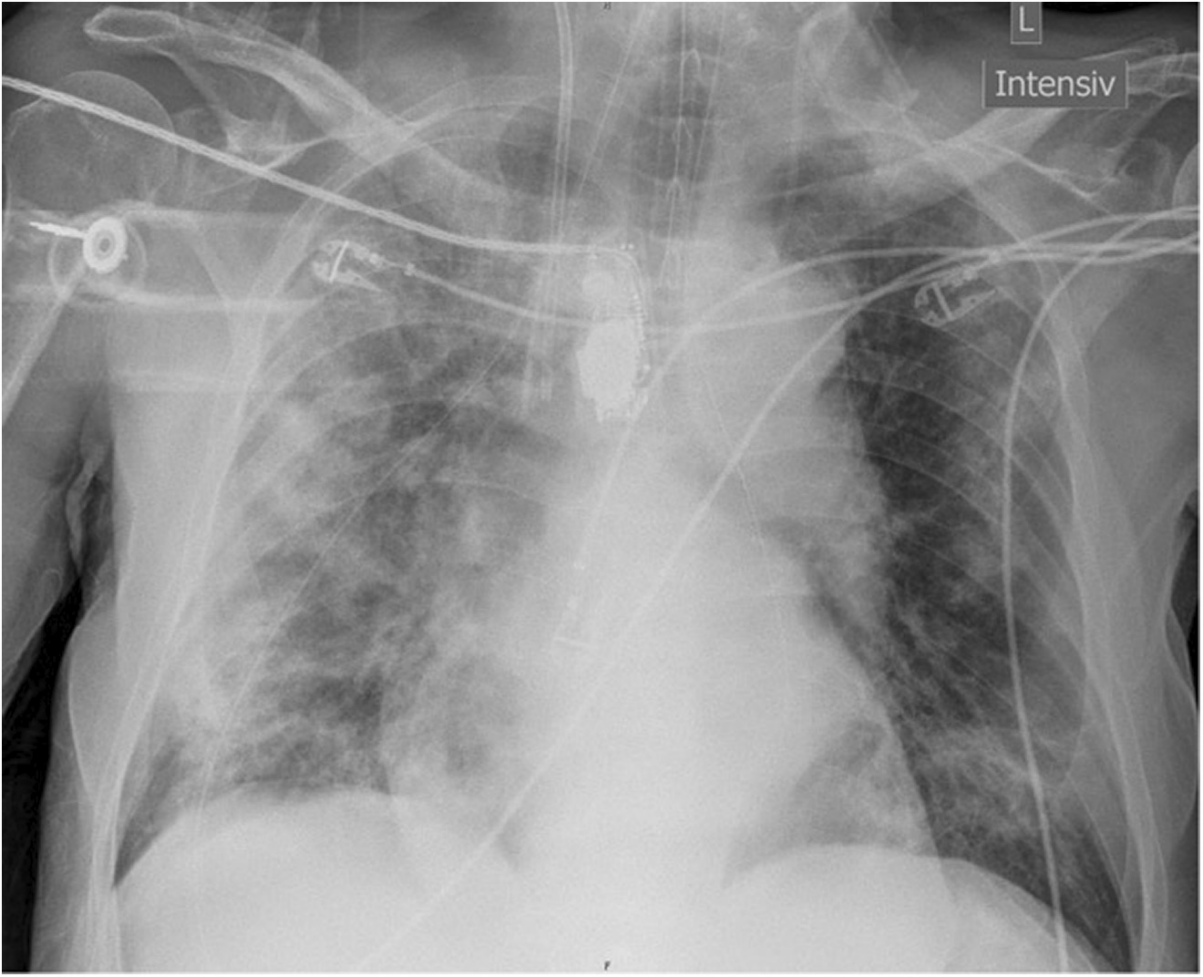
Chest radiography showed Covid‐19 typical bipulmonary infiltrates, which already occupied 70% of the patient's lung parenchyma. Chest radiography showed bipulmonary infiltrates typical of Covid‐19, which affected 70% of the lung parenchyma of the 90‐year‐old patient already on the day of admission to the ICU (day 2).

He was transferred to the intensive care unit on the second day after admission to the hospital. There, SpO_2_ was 88% with a respiratory rate of 40/min and a paO_2_ of 58.8 mmHg under HFNC 90% FiO_2_ and 50 L/min. Both, chest radiography and native CT chest radiography showed bi‐pulmonary infiltrates that already occupied 70% of the lung parenchyma and increased rapidly during the course (Figure 1). The patient was sedated and intubated, then dorso‐ventral alternate positioning was initiated. In the first blood gas analysis after intubation, a paO_2_ of 80.6 mmHg with a Horovitz factor of 89.5 mmHg was detected under a FiO_2_ of 90%, PEEP of 10 mbar, and a SpO_2_ of 95% under mechanical ventilation. Alternate positioning was continued for 7 days, and invasive ventilation was maintained for 16 days overall. However, the intensive care stay was not without complications. An unsuccessful attempt to insert a central venous catheter (CVC) and a Shaldon‐hemodialysis‐catheter into the left internal jugular vein resulted in the development of an ipsilateral pneumothorax on the same side; this mantle pneumothorax was treated conservatively and resorbed without consequence. An attempt at dilated tracheostomy was unsuccessful due to anatomic reason, and the patient was eventually extubated successfully, 16 days after intubation.

Therapy with dexamethasone, already initiated in the emergency department, was continued for a total of 8 days; administration of immune plasma was not indicated because the patient already had an immunocompetence of 281 AU/mL SARS‐CoV2 IgG after vaccination, which increased to 722 AU/mL by the time of discharge. The initial cycle‐threshold (CT) of the RT‐PCR was 20.5 and increased to 38.1 twenty‐one days after admission to the hospital. Anticoagulation was performed with heparin, and a target PTT of 50–60 s was aimed for.

**FIGURE 2:**
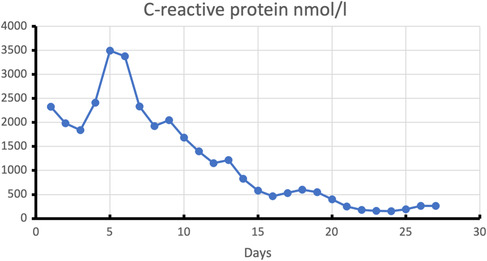
Development of C reactive protein values during hospitalization. C reactive protein (CRP) measurement in nmol/l shown on the y‐axis over the individual days (x‐axis), starting with the first day of hospitalization. Initiation of antibiotic therapy with piperacillin/tazobactam on Day 5, initiation of therapy with linezolid on Day 14.

**FIGURE 3:**
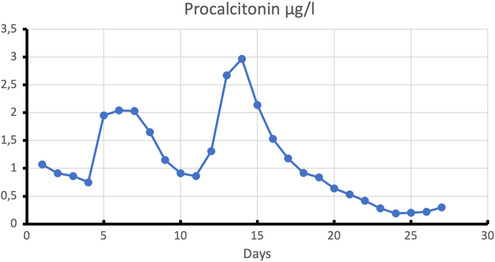
Development of procalcitonin values during hospitalization. Procalcitonin (PCT) measurement in μg/l shown on the y‐axis over the individual days (x‐axis), starting with the first day of hospitalization. Initiation of antibiotic therapy with piperacillin/tazobactam on Day 5, initiation of therapy with linezolid on Day 14.

Bacterial superinfection with *Escherichia coli* was detected in tracheal secretions and regressed with antibiotic therapy with Piperacillin/Tazobactam. Central line bloodstream infection caused by *Streptococcus epidermitis* was treated with Linezolid as tested. The time course of the infection parameters C reactive protein (CRP) and procalcitonin (PCT) under anti‐infective therapy are shown in Figures 2 and 3.

**FIGURE 4:**
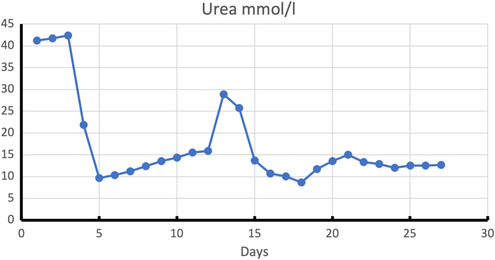
Development of urea levels during hospitalization. Measurement of urea in mmol/l on the y‐axis, charted over the individual days (x‐axis), starting with the first day. Sufficiency calculated fluid therapy until Day 5. Thereafter, renewed increase with subsequent dialysis requirement from Day 13 to 18.

**FIGURE 5:**
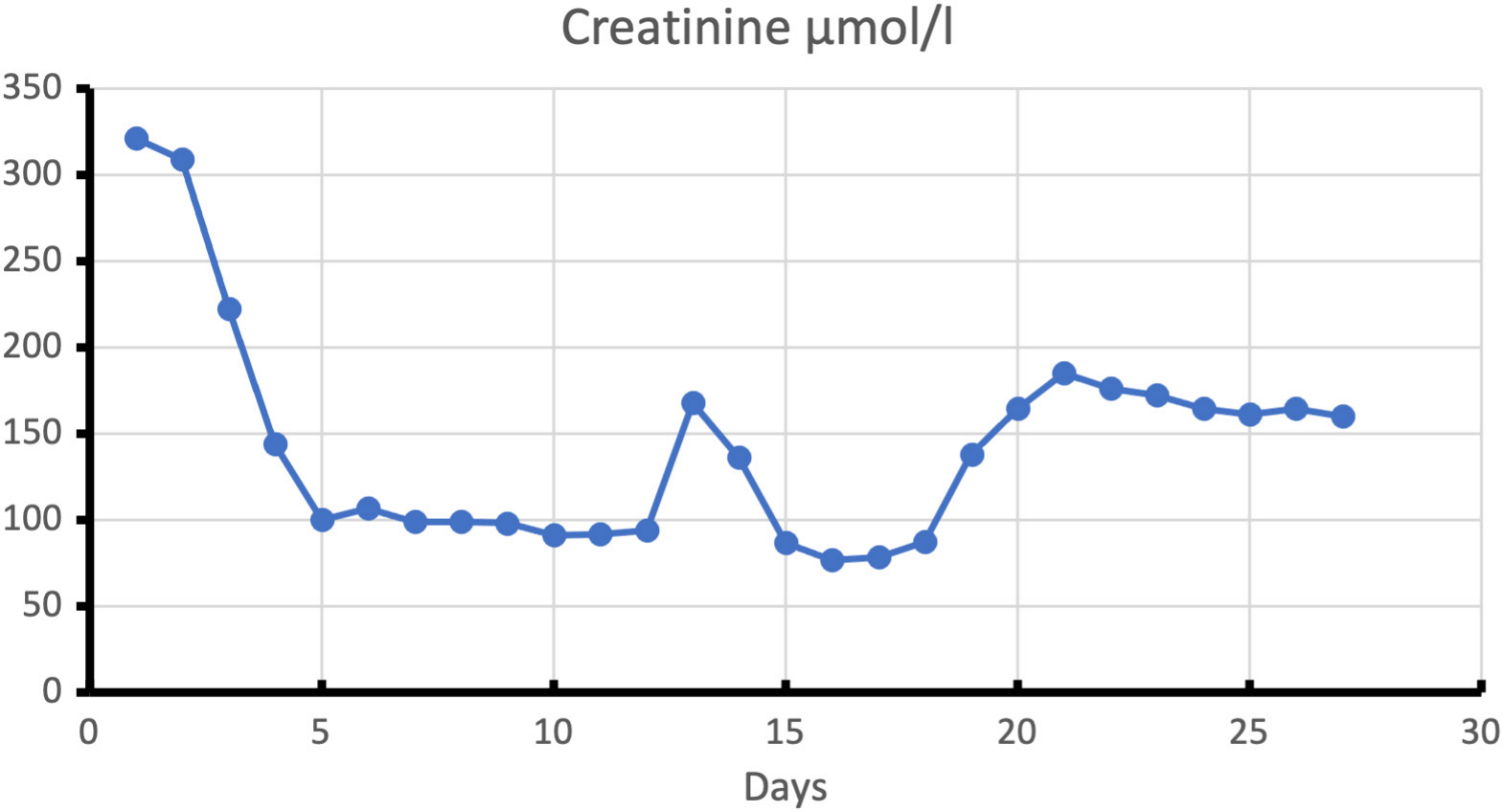
Development of creatinine values during hospitalization. Measurement of creatinine in μmol/l on the y‐axis, plotted over the individual days (x‐axis), starting with the first day. Sufficiency calculated fluid therapy until Day 5. Thereafter, renewed increase with subsequent dialysis requirement from Day 13 to 18.

The patient also suffered from an acute prerenal renal failure based on a pre‐existing chronic renal insufficiency. The reason for this exacerbation was immense fluid loss due to diarrhea and vomiting associated with the COVID‐19 infection. Initial fluid therapy was able to improve renal function. However, due to continually worsening pulmonary function from COVID‐19, fluid intake had to be kept restrictive as the disease progressed. This again led to exacerbation of the acute renal failure, which was successfully treated temporarily for 5 days with continuous veno‐venous hemodialysis (cvvHD) under calcium citrate anticoagulation. After this adjuvant therapy, urea and creatinine again showed the same values as before the COVID infection (Figures 4 and 5).

Forty‐six days after admission to the hospital and 28 days after extubation the patient could be transferred from intensive medical therapy to geriatric rehabilitation. From there, the patient was transferred back to his nursing home after additionally 21 days. The Barthel index was increased from 5 to 60 points during rehabilitation, which corresponded to the patient's approximate baseline score. The patient is living again in his facility with the same quality of life than before his COVID‐19 disease.

## 3 DISCUSSION

In this case report, we describe the medical history of a 90‐year‐old man who suffered from severe COVID‐19, caused by the SARS‐CoV2 variant B1.1.7. He additionally suffered several severe complications during hospitalization, and had multiple age‐related pre‐existing conditions. Due to peripartum rhesus incompatibility reaction, he also suffered from infantile hypoxic brain injury.

Since the last years, the medical possibilities in intensive care medicine have expanded remarkably and, of course, it can and must be discussed to what point which therapy is useful in each individual case. Often the opinions of the treating physicians, the nursing staff but also the relatives are diametrically opposed. Despite the ability to express their preference, more than 70% of seriously ill hospitalized elderly patients do not discuss their preferences with the healthcare provider. However, especially very elderly patients are rarely asked by the medical staff about their prior wishes and preferences. This might result in medical orders or invasive life‐sustaining therapies which are incongruent with the patients previously stated wishes and preference because the focus is deviated from comfort and quality of life.^12^, ^13^, ^14^ Of course, ICU admission may fail to improve or even worsen survival and/or quality of life.^12^, ^15^ This sometimes results in skepticism among the treatment team about admitting very elderly patients to the intensive care unit and treating them with the full spectrum of invasive intensive therapy. In very elderly patients, the fear of a deterioration in their quality of life and the thought of being a burden to their relatives also play a potential role in their personal decision‐making. It is reported that patients who were older or more severely frail were more likely to die, less likely to be admitted to critical care, and more likely to require higher care levels on discharge in survivors.^16^ There is a dilemma here that is difficult to resolve, as on the one hand not being admitted to the intensive care unit can of course significantly increase the mortality rate, especially in the case of pneumonia, but on the other hand invasive intensive care medicine can lead to complications and side effects, especially in older patients with a high degree of frailty negatively affect the further course of the disease.^8^

The aim of intensive therapy is to restore organ function that is threatened or lost due to illness and to bridge the organ function restriction with intensive therapy until its function is restored. Of course, especially in the case of severe disease progression, residuals can occur due to the disease itself or due to the necessary invasive therapy measures with side effects and complications, which have a negative impact on the quality of life. In older patients in particular, however, there is often a willingness to accept functional limitations. In an interesting study 20 years ago, Graf and colleagues tracked the quality of life of 153 patients with cardiovascular and pulmonary diseases after intensive care. They determined the quality of life 1 week before admission, 1 month after discharge and 9 months after discharge using the Medical Outcome Survey Short Form‐36 (SF‐36). Irrespective of the severity of illness and organ dysfunction at admission, quality of life had returned to at least baseline values in all patients after 9 months.^17^ In another study on quality of life and the individual costs, the same working group was able to show that 190 of 303 patients with cardiovascular and pulmonary diseases were still alive 5 years after initial intensive care therapy and 173 of whom completed the SF‐36 questionnaire. About 79% of the former intensive care patients continued to live independently in their own household after the 5 years period, 9% lived with support in their own household and 12% lived with relatives or in a nursing home. During the 5 years follow‐up, 100 of the patients were admitted to a hospital once (40%) or several times (60%). The functional status and the quality of life experienced by the majority of patients surviving the ICU were reasonably good.^18^

Another secondary result of the study is particularly interesting with regard to the above‐mentioned skepticism about intensive care in older patients. Only 36 patient (21%) recalled pleasant or dreadful memories toward the ICU stay, 25 (15%) had no memories, 68 (39%) had neutral memories and 44 (25%) had pleasant memories. The majority of patients (91%) would—if necessary—approve ICU admission again.^18^

One of the most important challenges for the intensivist is the allocation of scare intensive resources to those patients who will benefit from intensive care therapy.^18^ This challenge is even greater in a pandemic.

In general, admission to the intensive care unit requires, first, the patient's desire for treatment and, second, the medical necessity and meaningfulness of the treatment. However, there is still no pre‐emptive score for any severe disease that can reliably predict the intensive care outcome in an individual patient. Therefore, the intensive care physician can only talk about the probability of occurrence of both positive aspects such as a good outcome and negative aspects such as complications and side effects. But it seems all the more important to talk to our patients or their relatives in order to find out what the patient wishes and preferences are. Even patients and their relatives should ideally discuss in advance of a life‐threatening illness if in occurrence of a severe illness invasive intensive care or comfort care is desired.^12^

Luckily, in this case, the patient's wish was clearly stated by the niece who looked after him. As, almost without exception, most countries around the world did not have clear legal guidelines for prioritizing limited intensive care resources at the time of our patient's admission, this case was handled according to the wishes of the patient and his relatives. Although based on the severity of the COVID‐19 disease, the age, the previous illnesses and the frailty, in this case a negative outcome of the disease was more likely. We cannot judge if the previous repression in his early childhood probably played an unconscious role in the decision making of the medical staff. However, during the entire stay in intensive care, despite various setbacks and complications, no therapy limitations or changes in the therapy goals were considered. The positive outcome of the patient after this long and difficult course of the disease was correspondingly gratifying for the medical treatment team.

In the course of the pandemic, some countries have issued guidelines on how to proceed in the event of triage. In the UK, for example, a guideline was developed by the National Institute for Health and Care Excellence that recommended the use of the clinical frailty score for all patients on admission.^19^ The use of CFS was also recommended in Sweden during the pandemic.^20^ During the initial phases of the pandemic, the use of CFS was also recommended in Canada^16^ and Australia.^21^

Fortunately, there are now clearer guidelines in many countries, such as Germany, for a possibly necessary priority situation in the context of a resource imbalance. Here, for example, the allocation decision may only be made on the basis of the current and short‐term survival probability of the patients concerned, irrespective of disability, age, remaining life expectancy, and other characteristics.^22^

Several retrospective analyses showed in patients tested positive for SARS‐CoV‐2 that higher age and frailty are associated with a higher in‐hospital mortality.^8^, ^20^ Hägg and colleagues reported data from geriatric patients admitted to a large Swedish hospital with a diagnosis of COVID‐19. Older age, CFS >5, a higher Charlson comorbidity index (CCI) and acute kidney injury were associated with a higher in‐hospital mortality and the first two items were further associated with a lower probability of being discharged home. The authors concluded that 76% of the hospitalized geriatric patients with COVID‐19 survived, indicating that providing hospital level care to frail older patients with this disease is not futile.^20^ Certainly, the clinical frailty scale (CFS) is an aid for preoperative prophylaxis and perioperative treatment and it can also be an aid for therapy limitation in case of bad course of the critical illness.^23^, ^24^, ^25^ Hubbard and colleagues agreed in a worth reading commentary that a multidimensional measure of frailty such as the CFS has value in allocation of scarce health resources. However, they pointed out that it is important for clinicians and administrators to understand its limitations when used in the acute hospital setting. Frailty is not synonymous with end‐of‐life.^21^ In summary, Hubbard and colleagues recommend against the use of screening (including the CFS when used as such) as the sole component to ration access of older people to health care. Instead of this they recommend that frailty screening tools are implemented as a rapid component of a person‐centred approach to assessment that takes account of three key biomedical factors: severity of the presenting acute illness, the likelihood of medical interventions being successful, and the degree of frailty.^21^

The CFS can be used in particular together with a comorbidity assessment to identify the risk of a severe course of COVID‐19 disease in older patients in order to then provide these patients with multi‐dimensional and effective medical care and interventions in order to ultimately improve the outcome of this particularly vulnerable group.^8^, ^20^

## 4 CONCLUSION

This case report can be seen as a first confirmation of Holm and Warrington's thesis^11^ that the frailty index seems inappropriate both for patients who have suffered from physical and mental disabilities for many years and as a triage tool in a pandemic. Limitative decisions must always be made in plenary (physicians, nurses, and family members) for the individual patient. Vital signs and scores can give us a guideline, but one should not rely on them and base one's decision purely on these methods of measurement. Fortunately, many countries now have national guidance on the priorities that may be required in the context of a resource imbalance. Certainly, in another pandemic, when we are faced with the decision of which patients not to treat again, we will need reliable tools, but for this reason we have to keep searching and researching for more reliable tools.

## AUTHOR CONTRIBUTIONS

**Andreas Wehrfritz:** Conceptualization; data curation; writing – original draft; writing – review and editing. **Joachim Schmidt:** Data curation; investigation; writing – original draft; writing – review and editing. **Frank Bremer:** Formal analysis; validation; writing – original draft; writing – review and editing. **Anne‐Katharina Lang:** Conceptualization; data curation; writing – review and editing. **Jacob Welzer:** Conceptualization; data curation; writing – original draft. **Ixchel Castellanos:** Formal analysis; supervision; validation; writing – original draft; writing – review and editing.

## FUNDING INFORMATION

We acknowledge financial support by Deutsche Forschungsgemeinschaft and Friedrich‐Alexander‐Universität Erlangen‐Nürnberg within the funding programme “Open Access Publication Funding.”

## CONFLICT OF INTEREST STATEMENT

The authors declare that they have no financial or personal conflicts of interest associated with this case report.

## ETHICS STATEMENT

The ethical approval was not required for the case report.

## CONSENT

Written informed consent was obtained from the patient's caregiver (niece) for publication of this case report.

## Data Availability

The data that support the findings of this study are available from the corresponding author upon reasonable request.
